# Modifying Pavlovian-to-instrumental transfer by approach avoidance training in healthy subjects: a proof of concept study

**DOI:** 10.1038/s41598-023-37083-3

**Published:** 2023-06-21

**Authors:** Annika Rosenthal, Ke Chen, Anne Beck, Nina Romanczuk-Seiferth

**Affiliations:** 1 Department of Psychiatry and Neurosciences|CCM, Charité–Universitätsmedizin Berlin, Corporate Member of Freie Universität Berlin and Humboldt-Universität zu Berlin, Berlin, Germany; 2 Faculty of Health, Health and Medical University, Campus Potsdam, Potsdam, Germany; 3 Department of Psychology, Clinical Psychology and Psychotherapy, MSB Medical School Berlin, Berlin, Germany

**Keywords:** Human behaviour, Cognitive neuroscience, Cognitive control, Learning and memory, Classical conditioning

## Abstract

The modulation of instrumental action by conditioned Pavlovian cues is hypothesized to play a role in the emergence and maintenance of maladaptive behavior. The Pavlovian to Instrumental transfer task (PIT) is designed to examine the magnitude of the influence of cues on behavior and we aim to manipulate the motivational value of Pavlovian cues to reduce their effect on instrumental responding. To this end, we utilized a joystick-based modification of approach and avoidance propensities that has shown success in clinical populations. To examine changes in PIT, we subjected 35 healthy participants to a series of experimental procedures: (1) Instrumental training was followed by (2) Pavlovian conditioning of neutral stimuli that were associated with monetary reward or loss. (3) In a subsequent joystick task, approach and avoidance tendencies toward conditioned cues were assessed. (4) In a transfer test, the PIT effect as the impact of conditioned cues on instrumental behavior was measured. (5) The explicit knowledge of cue-reward contingencies was assessed in a forced-choice phase. (6, 7) systematic joystick training was followed by a posttest (8) the transfer task and forced-choice test were repeated. We found no effect of training on approach-avoidance propensities in the context of this proof of concept study. A higher response rate towards negative stimuli during PIT after systematic training compared to sham training was seen. On the other hand, we saw an increased PIT effect after sham training. These results contribute to the understanding of the strength of the influence of cues on instrumental behavior. Our findings further stress the importance of context, instructions and operationalization of instrumental behavior in the framework of transfer effects.

## Introduction

Stimuli that are associated with rewards have been demonstrated to encourage behaviors attributed to past rewarding experiences^1,2^. This so-called concept of cue-reactivity is central to human and animal adaptive behavior such as food seeking or reproduction^2^. Cue-reactivity entails that our actions are continuously influenced and guided by predictive cues, which can prompt or deter us from engaging in certain behaviors. As stated above, these cues can be either adaptive or maladaptive, leading to suboptimal choices. However, cue-reactivity has been mostly researched in the background of disparaging behavior such as substance abuse, binge eating, or other behavioral patterns marked by conflicts of behavioral goals and values assigned to stimuli^3–5^. For instance, after initially producing rewarding effects, prolonged drug abuse could alter motivational drive and sensitize to drug-related conditioned responding and craving^6,7^. These factors play a fundamental role in the maintenance of substance use disorders (SUDs). The interplay of instrumental behavior and reinforcing properties of stimuli has been conceptualized in various ways. One way cue-motivated behavior has been modeled, is through Pavlovian-to-instrumental transfer (PIT) tasks^8,9^. The earliest studies on PIT can be traced back to the 1940s. These studies revealed that stimuli that were associated with food, could increase the likelihood of instrumental actions directed towards food^9^. Transfer effects can have both facilitating and inhibiting effects on actions, as cues can either increase or decrease the frequency of certain actions or influence preference towards specific actions. The direction of transfer effects depends on several factors, such as the valence of the Pavlovian cue, which refers to whether it is appetitive or aversive. For instance, a Pavlovian cue linked to an aversive shock may encourage actions aimed at avoiding shock but discourage actions aimed at obtaining food (Rescorla and Solomon, 1967). This action-specific control of behavior has been shown and researched in humans as well^10–12^. Essentially, PIT tasks constitute instrumental training to establish response–outcome associations by linking responses to reward delivery. In a Pavlovian conditioning phase, previously neutral stimuli (CS) predict rewarding outcomes (US), to establish stimulus–outcome CS–US) associations. In the transfer test, instrumental behavior is assessed in extinction and in presence of the outcome-associated stimuli (CS-US)^13^. An increase of instrumental behavior due to high motivational salience (i.e., preferential attentional processing) of the Pavlovian CS has been found in various clinical populations as well as at-risk groups, i.e. alcohol use disorder^14^, social drinking^15^, obesity^16^ as well as aversive PIT was exaggerated in patients with depression^17^, while some also found transfer effects to be reduced in patients with depression^18^. In addition, increased loss aversion PIT was found in subjects with gambling disorder^19^. Despite its’ importance in psychopathology, the influence of environmental cues on behavior is pertinent to decision-making and instrumental choice behavior in general. Accordingly, PIT effects are a phenomenon that has been researched in healthy populations^20^. Nevertheless, the underlying mechanisms of the nature of transfer effects are debated^21^. Associative accounts postulate the dissociative engagement of motivational and cognitive control and within this context, biased action selection towards reward cues has been explained^22^. Similarly originating within a dual-process framework, that divides cognitive function into implicit and automatic or explicit and controlled processes, cognitive bias modification (CBM) was developed with the idea to evaluate these biases in the context of maladaptive behavior^23^. One form of CBM focuses on bias in the automatically activated action tendency to approach or avoid certain stimuli. This approach avoidance task (AAT) operationalizes push (avoid) and pull (approach) behavior with a joystick experiment in which subjects are instructed to react to content or content-unrelated features of a stimulus. Studies have shown that an increased approach bias towards drug-related stimuli was related to consumption or addiction severity^24,25^ as well as food associated stimuli increased approach bias in food craving^26^ and was correlated to uncontrolled eating^27^. In this context, modified versions of the AAT have been used to successfully retrain these altered approach/avoidance tendencies in clinical populations^28–30^. An indication of efficacy of AAT training is uncertain, however, due to mixed results for reviews, see^31,32^. In extension of this, Pavlovian conditioning of previously neutral cues has been shown to elicit significant approach tendencies towards these cues as well. For example, approach bias was enhanced when participants were faced with abstract stimuli that were previously paired with chocolate^33^ or tobacco^34^.

Regarding the role of implicit motivational processes that drive behavior, PIT effects and approach bias have been theorized to influence each other but the exact nature of this has not been disentangled yet. Both concepts have been shown to overlap in salience attribution of external cues, ultimately driving behavior. This is known as e.g., incentive salience in the context of clinical disorders^35^*.* While it has been proposed that approach bias plays a role in PIT^36^, it has also been stipulated that transfer effects drive approach bias^37^. In addition, research suggests that the PIT and AAT are associated on a neuronal and behavioral level^38^. Considering clinical relevance, increased PIT effects in psychiatric populations, especially in the addiction domain, have not been subjected to systematic modification yet. It has been proposed, however, that clinical populations with strong PIT effects could profit from approach modification training to reduce PIT effects and increase behavioral control^14^. From a therapeutic perspective, it makes sense to systematically reduce the effect of Pavlovian cues that trigger maladaptive behavior: on the one hand, to prevent possible situational habit formation in at-risk populations and on the other hand, to disrupt the effect of environmental cues and ensure abstinence from already established dysfunctional behavior.

While the effect of alcohol-related AAT training on PIT effects has been investigated^39^, the direct manipulation of experimentally Pavlovian conditioned stimuli has not been attempted thus far. In light of this, we want to investigate whether PIT effects can be translated into approach and avoidance biases towards previously conditioned cues in the context of an AAT and subsequent retraining of these biases. To examine this in a healthy population, we will employ a previously established task that uses monetary cues^40^. We first want to evaluate whether Pavlovian conditioning of neutral cues in the context of a PIT task elicits approach and avoidance biases toward them. In a next step, we want to find out whether these action tendencies can be manipulated via a modified training version of the AAT. Lastly, we want to assess the effects of this systematic manipulation of the approach avoidance propensities of Pavlovian cues on Pavlovian-to-Instrumental transfer (PIT) processes in healthy adults.

## Methods

All procedures complied with the Declaration of Helsinki and were approved by the ethical committee of the Charité–Universitätsmedizin Berlin. All participants gave full written informed consent.

### Participants

The study was conducted in Berlin, Germany and all participants were recruited through internet advertisement. Participants were included if between 18 and 65 years of age and they were neither pregnant nor breastfeeding. To exclude subjects with pathological manifestations of traits that could confound our results, exclusion criteria were personality disorders, lifetime bipolar disorder, acute depressive episode, and SUD (except tobacco use disorder (TUD) and mild (up to 6 criteria) cannabis use disorder (CUD)) according to the Diagnostic and Statistical Manual of Mental Disorders (Fifth Edition) (DSM-5). The sample consisted of 35 participants (22 female) and age ranged from 19 to 60 (mean = 35.9 SD = 12.01) (*see *Table 1*.*).

**Table 1:** Descriptives of sham- and intervention training group.

	Sham training	Intervention training	*p/x*^*2*^
	*Mean (SD)*	*Mean (SD)*	
Age	37 (11.86)	35.15 (12.45)	0.66
	*Male (female)*	*Male (female)*	
Gender	6 (9)	7 (13)	0.76

*X*^*2*^ chi-squared, *SD* standard deviation.

### Procedure

**Figure 1:**
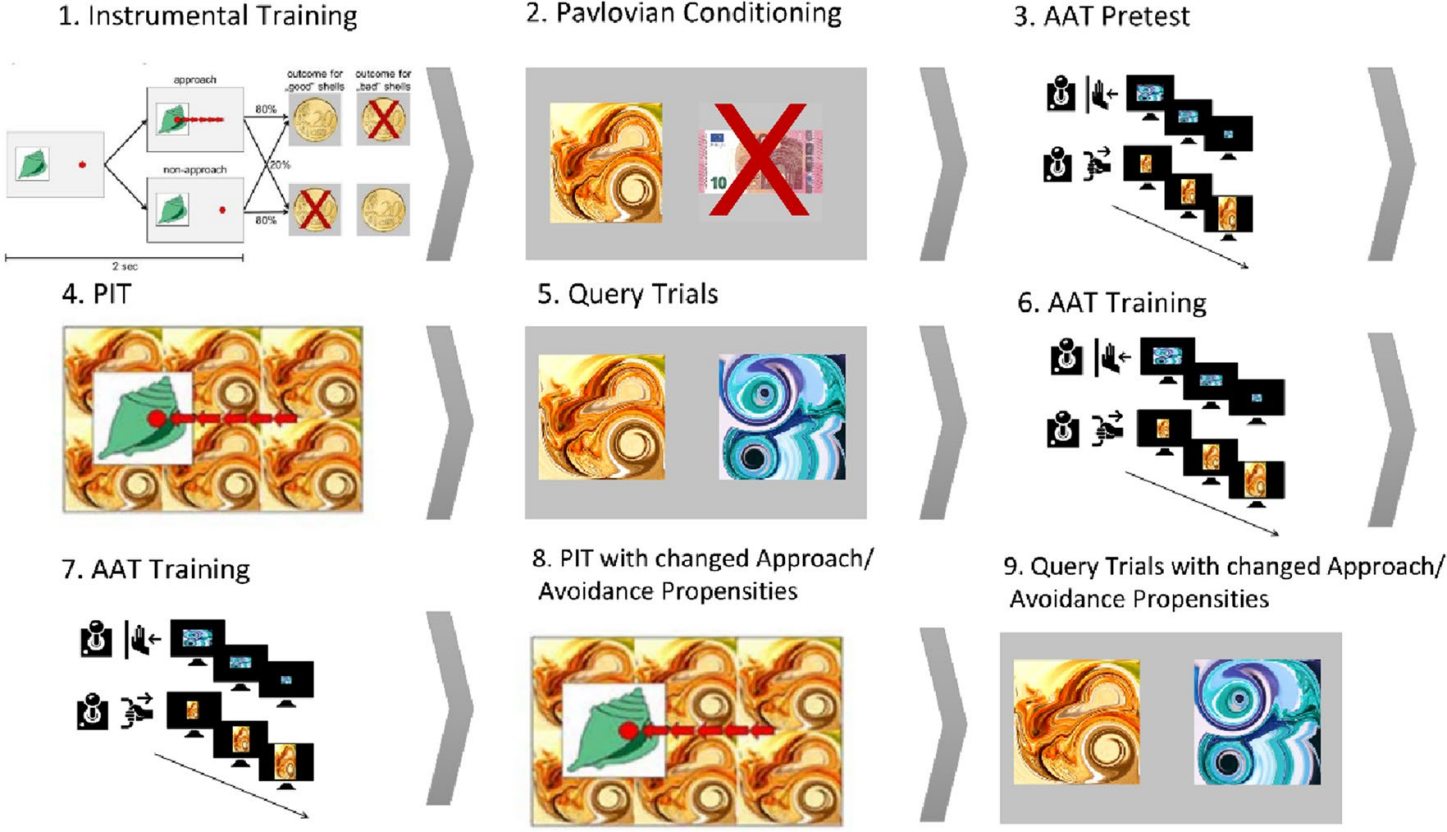
Schematic representation of the study design. Instrumental training (**1**) and Pavlovian conditioning (**2**) is followed by an AAT pretest (**3**). After PIT and Query trials (**4,5**), AAT training (**6**) and posttest (**7**) are followed by a second PIT and Query trials (**8,9**). AAT approach avoidance training, PIT Pavlovian-to-Instrumental transfer.

The PIT paradigm was administered in six parts, which consisted of (1) instrumental training, (2) Pavlovian training, (3, 5) PIT before and after AA training, and (4, 6) a forced choice task before and after AA training (see Fig. 1A–F). The task was programmed with Matlab 2019b (MATLAB version 9.7.0, 2019; MathWorks, Natick, MA, USA) using the Psychophysics Toolbox Version 3 (PTB‐3.0.15) extension^41–43^. For an extensive description of the PIT paradigm, please see Garbusow *et al*.^40^. After the Pavlovian conditioning phase, we administered the AAT to assess the participants' approach/avoidance bias. Following PIT and the forced choice task, the AAT training and post-test were administered (please see Fig. 1 for details of the study design).

### Instrumental training

Instrumental stimuli consisted of six shells in various colors and shapes that were presented on a computer screen. The instructions to the participants were to collect “good” and leave “bad” shells while receiving probabilistic feedback. In order to collect a good shell, the subjects had to repeatedly press the left mouse button (at least 5 times), while they had to omit a reaction when a bad shell was presented (0–4 button presses were counted as omission in order to measure vigor). In a random fashion, correct responses were rewarded with 20 Cents in 80% of the trials and punished with a loss of 20 Cents in 20% of trials, and vice versa for incorrect responses. Dependent on performance, a learning criterion determined the task length to be between 60 and 120 trials (80% correct trials over 16 consecutive trials).

### Pavlovian conditioning

Subjects were presented with 48 trials of abstract image sound combinations (compound CS) that were paired with monetary reward or punishment (US) in a deterministic fashion. The compound CS was presented for 3 s on the left or right side of the screen, followed by a delay of 3 s with two fixation crosses at the two potential CS locations, then a US (monetary reward 10€, punishment -10€ or no stimulus) was presented for another 3 s. The comparatively large amount of money is intended to increase the influence of Pavlovian conditioning, while the small amount is intended to elicit variable accuracy in the context of instrumental training. Subjects had to passively watch and memorize the CS and US pairings. Abstract pictures and associated US were randomly paired.

### Pavlovian‐to‐instrumental transfer

The instrumental task was performed in formal extinction, which means that the monetary outcome was not shown. During the trials, the background was alternately tiled with one of the CS. The task had a duration of 108 trials with each lasting 3 s.

### Query trials

To avoid mixing Pavlovian and instrumental conditioning, a forced choice task was administered after the transfer stage to confirm the success of the Pavlovian conditioning. Based on their subjective preference, subjects had to choose between two Pavlovian CSs (9 trials). All pairings were presented in an interleaved, randomized order.

### AAT

**Figure 2:**
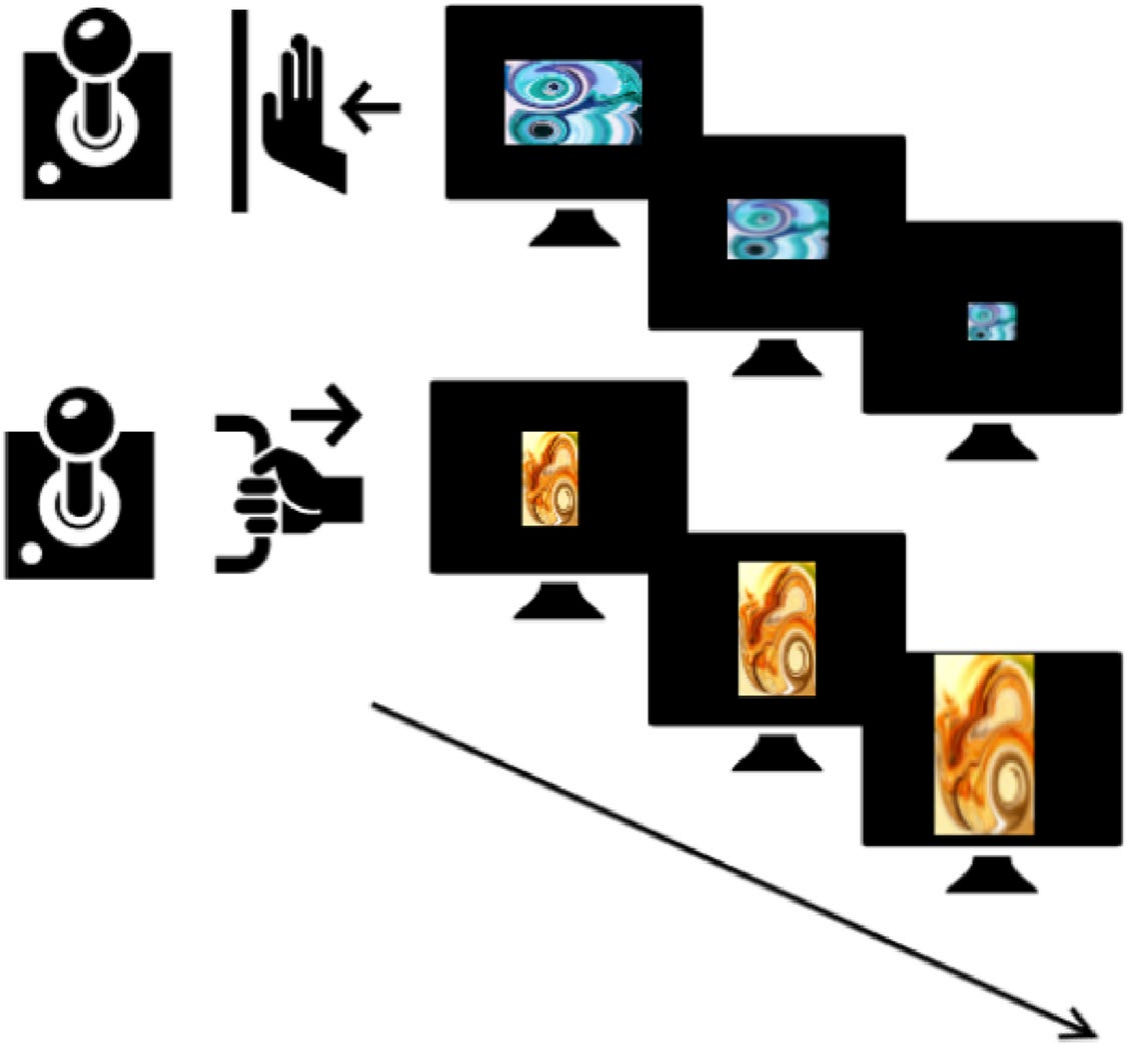
In detail representation of the AAT training. Positively conditioned fractals are pushed away (top) and negatively conditioned fractals are pulled towards the subject (bottom). *AAT* approach avoidance training.

This version of the AAT was programmed so that participants had to respond according to the orientation of the pictures (*see *Fig. 2). With a joystick, all horizontal pictures were to be pushed away (avoidance), and all vertical pictures were to be pulled closer (approach). To mimic realistic approach/avoidance behavior, a zoom feature was used. In the approach (pull) movement, pictures grew larger; while pushing resulted in shrinking pictures. The task structure was adapted from Wiers et al.^44^. As stimuli, we used the same abstract pictures (CS) as in the PIT task.

The AAT consisted of three different phases: a pre-test AAT, a training AAT, and a post-test AAT. A practice phase of 40 trials, in which participants learned to move the joystick according to orientation, was followed by 120 trials in which all three abstract pictures had to be pushed/pulled with equal frequency. During the training phase which consisted of 300 trials, participants moved the joystick according to the assigned condition. In the sham condition, positive and negative stimuli were shown equally often in push and pull trials. In the intervention condition, participants had to push positively conditioned pictures (10€) and pull negatively conditioned pictures (− 10€) while neutral pictures were both pushed and pulled with equal frequency. In the post-test phase, 120 trials with equal frequency of all possible trial types were administered.

### Analysis

#### Statistical analysis plan

Data were analyzed in Matlab 2011a and Jamovi (The Jamovi Project, 2022). Generalized linear modeling (GLM), as well as generalized linear mixed modeling (GLMM), was used since the RT data from the AAT were non-normally distributed (Kolmogorov–Smirnov: p < 0.001) and the PIT response showed a zero inflation as no response was required on some of the trials. All models used log link functions. The full fixed effects structure of the model was supplemented by the step-wise inclusion of random effects while testing their respective contribution to the model fit. Fixed effects and their interactions were tested using omnibus (Wald) Chi-Squared tests (results are reported in the supplementary Tables 1–4).

### AAT

To exploratorily assess the individual contribution of Pavlovian CS on approach avoidance behavior at pre and post training, initial gamma distributed models were applied to test for the effect of background CS (− 10€/neutral/+ 10€; dummy coded with − 10€ as a reference), direction (push/pull; coded as − 0.5/+ 0.5) as well as their interaction on RT in each trial. Subsequently a GLMM, a gamma distributed model was built in which the RT in each trial was predicted by the value of the background CS (− 10€/neutral/+ 10€; dummy coded with − 10€ as a reference) the direction (push/pull; coded as − 0.5/+ 0.5), the training condition (Sham/Avoidance; coded as − 0.5/+ 0.5) and time (pre Training/post Training; coded as − 0.5/+ 0.5) as well as their interactions. Initial model comparison of different random-effects structures indicated the best model fit for taking intercept, main effects of CS value, direction, and time as random effects across subjects. Please see supplementary Table 5 for model comparison based on the Akaike Information Criterion (AIC)^45^ of different random-effects structures.

### PIT

Initial model fitting indicated a Poisson distributed model to be overdispersed (*Chi*^*2*^*/DF* = 3.77)^46^. To account for this, a model with negative binomial distribution was built to assess the individual contribution of Pavlovian values on behavior. Here, the number of button presses in each trial was predicted by the value of the background CS (− 10€/neutral/+ 10€; dummy coded with − 10€ as a reference) the instrumental condition (not collect/collect; coded as − 0.5/+ 0.5), the training condition (Sham/Avoidance; coded as − 0.5/+ 0.5) and time (pre Training/post Training; coded as − 0.5/+ 0.5) as well as their interactions. Model comparisons indicated the within-subject factors intercept, main effect of CS value, and time to be the best fitting random factor structure. Please see supplementary Table 6 for model comparison based on AIC of different random-effects structures.

## Results

Of the experimental trials, 6.92% were excluded (implausible RTs based on 1st and last percentile as well as all participants with commission errors > 21% based on^47,48^). The n = 1 excluded participant had error rates of 39.2% and 40% in the AAT pretest and posttest respectively.

### AAT

In the pre training AAT, we did not find a significant effect of the background CS, direction, or their interaction (supplementary Table 7/Fig. 1).

For summary statistics of AAT RT pre and post training conditions, please see Table 2.

**Table 2:** Descriptives of reaction time in the approach avoidance task (AAT), stratified by direction, time, training condition, and CS.

Direction	Time	Training condition	CS	RT	
				Mean	SE
Push	Pre	Sham training	− 10€	718.73	12.36
			0	692.55	10.70
			10€	708.87	11.89
		Intervention training	− 10€	699.69	9.27
			0	692.43	10.22
			10€	692.75	9.95
	Post	Sham training	− 10€	661.74	9.08
			0	659.85	8.78
			10€	654.71	7.59
		Intervention training	− 10€	659.62	9.80
			0	641.49	7.59
			10€	628.06	6.83
Pull	Pre	Sham training	− 10€	688.45	12.21
			0	690.46	10.30
			10€	682.31	9.50
		Intervention training	− 10€	706.13	12.01
			0	689.62	9.03
			10€	683.35	9.43
	Post	Sham training	− 10€	648.66	8.61
			0	666.07	10.76
			10€	659.27	9.87
		Intervention training	− 10€	644.90	6.97
			0	655.97	7.70
			10€	661.89	9.04

*RT* reaction time, *CS* conditioned stimulus, *SE* standard error.

**Figure 3:**
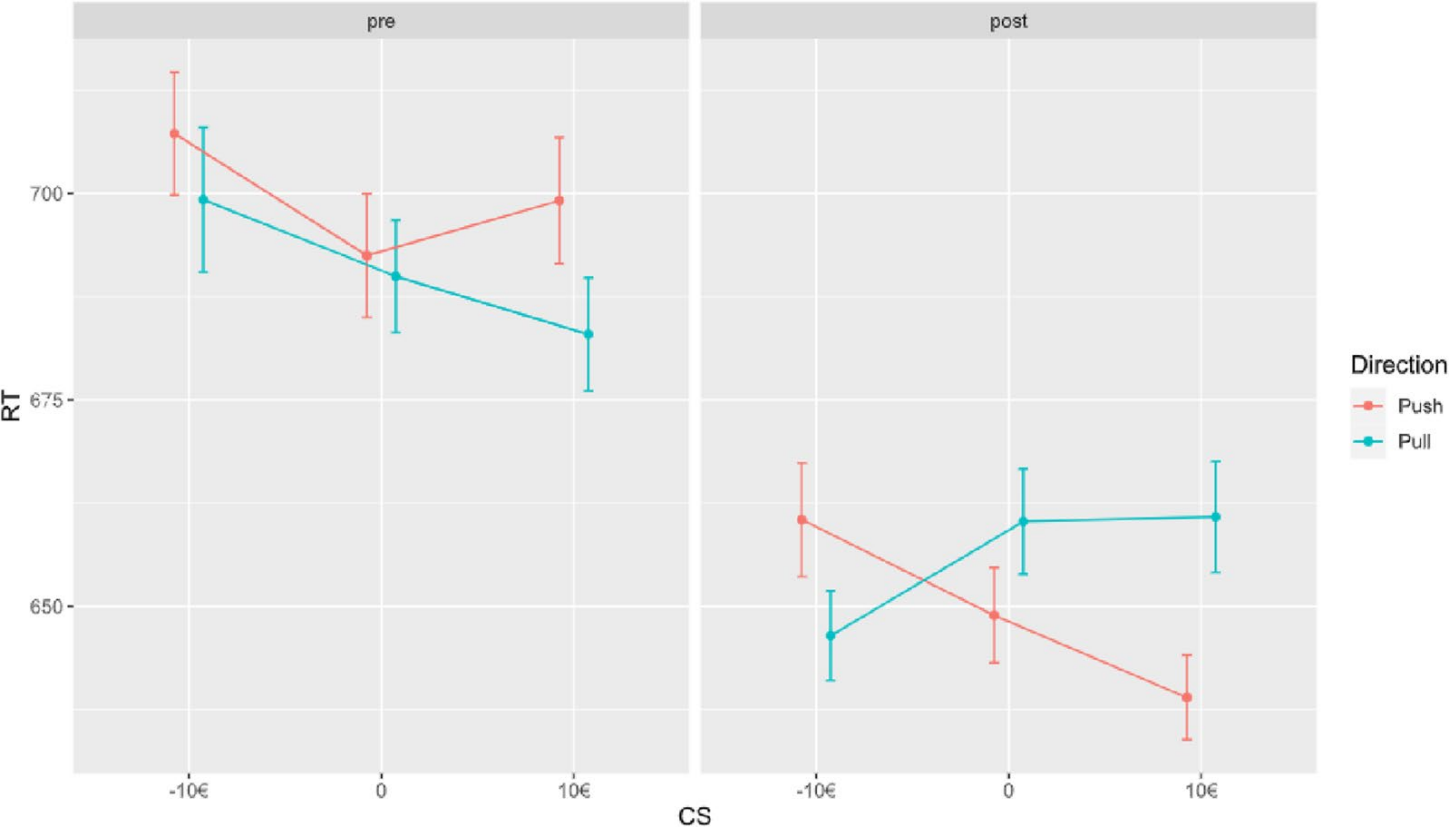
There was an overall effect of time, participants responded faster in the post AAT. In addition, after training, there was an increase in RT towards positive CS. *RT* reaction time.

In our GLMM, the main effect of interest was the interaction between training by time by CS by direction on RT. This however, was not significant (*estimate* = 0.061; SE = 0.041; *p* = 0.140), indicating there was no effect of training on the change of approach or avoidance bias towards the CS (*please see *Table 3* and *Fig. 3). However, there was a main effect of time (*estimate* = − 0.075; SE = 0.025; *p* = 0.003), indicating a general decrease of RT from pre AAT to post AAT. Moreover, the direction by CS interaction was significant (*estimate* = 0.025; SE = 0.010; *p* = 0.016). Additionally, the CS by direction by time interaction was significant (*estimate* = 0.051; SE = 0.021; *p* = 0.015), indicating that irrespective of training, the RT of approach of positive CS was significantly higher compared to approach RT towards negative CS. Please see Fig. 3 for a graphic representation of the results of this interaction.

**Table 3:** Results of generalized linear mixed model testing the interaction effect between response direction, conditioned stimulus, time, and training on reaction time in the AAT.

Fixed effects parameter estimates							
Names	Estimate	SE	95% Confidence Interval		exp(B)	z	p
			Lower	Upper			
(Intercept)	6.502	0.031	6.441	6.562	666.319	209.963	< 0.001
CS (10€–10€)	− 0.007	0.011	− 0.028	0.013	0.993	− 0.700	0.484
Direction (pull)	− 0.022	0.015	− 0.051	0.008	0.979	− 1.449	0.147
Time (post training)	− 0.075	0.025	− 0.124	− 0.026	0.928	− 2.979	0.003
Training (intervention training)	− 0.014	0.062	− 0.135	0.108	0.986	− 0.221	0.825
CS (10€–10€) ✲ direction (pull)	0.025	0.010	0.005	0.045	1.025	2.398	0.016
CS (10€–10€) ✲ time (post training)	0.008	0.010	− 0.012	0.029	1.008	0.809	0.418
Direction (pull) ✲ time (post training)	− 0.003	0.015	− 0.031	0.026	0.997	− 0.184	0.854
CS (10€–10€) ✲ training (intervention training)	− 0.013	0.021	− 0.054	0.028	0.987	− 0.612	0.540
Direction (pull) ✲ training (intervention training)	0.020	0.030	− 0.039	0.078	1.020	0.654	0.513
Time (post training) ✲ training (intervention training)	− 0.014	0.050	− 0.112	0.085	0.987	− 0.270	0.787
CS (10€–10€) ✲ direction (pull) ✲ time (post training)	0.051	0.021	0.010	0.091	1.052	2.437	0.015
CS (10€–10€) ✲ direction (pull) ✲ training (intervention training)	0.012	0.021	− 0.029	0.053	1.012	0.581	0.561
CS (10€–10€) ✲ time (post training) ✲ training (intervention training)	− 0.007	0.021	− 0.047	0.034	0.993	− 0.316	0.752
Direction (pull) ✲ time (post training) ✲ training (intervention training)	− 0.041	0.029	− 0.099	0.016	0.960	− 1.409	0.159
CS (10€–10€) ✲ direction (pull) ✲ time (post training) ✲ training (intervention training)	0.061	0.041	− 0.020	0.142	1.063	1.474	0.140

*CS* conditioned stimulus, *SE* standard error, *exp(B)* odds ratio.

### PIT

For the pre training PIT, we did find a significant effect of the background CS and instrumental response but no interaction (supplementary Table 4). As expected, subjects responded with more button presses in positive CS trials (mean = 4.30; SE = 0.154) than in neutral CS trials (mean = 3.68; SE = 0.134) and negative CS trials (mean = 3.05; SE = 0.114). In line with this, the effect of instrumental condition was significant as well (*estimate* = 1.192; SE = 0.047; *p* < 0.001), here button presses in collect trials were higher (mean = 6.19; SE = 0.175) than in not collect trials (mean = 2.14; SE = 0.670).

For summary statistics of PIT pre and post training conditions, please see Table 4.

**Table 4:** Descriptives of button presses in the Pavlovian-to-Instrumental task (PIT), stratified by training condition (pre/post AAT), time, and CS.

				Button presses	
				Mean	SE
Sham training	Pre AAT	CS	− 10€	3.150	0.179
			0	4.353	0.178
			10€	4.810	0.181
	Post AAT	CS	− 10€	3.258	0.189
			0	4.239	0.192
			10€	4.906	0.186
Intervention training	Pre AAT	CS	− 10€	3.613	0.165
			0	3.984	0.164
			10€	4.579	0.166
	Post AAT	CS	− 10€	3.933	0.167
			0	4.032	0.166
			10€	4.488	0.169

*CS* conditioned stimulus, *SE* standard error, *AAT* approach avoidance training.

**Figure 4:**
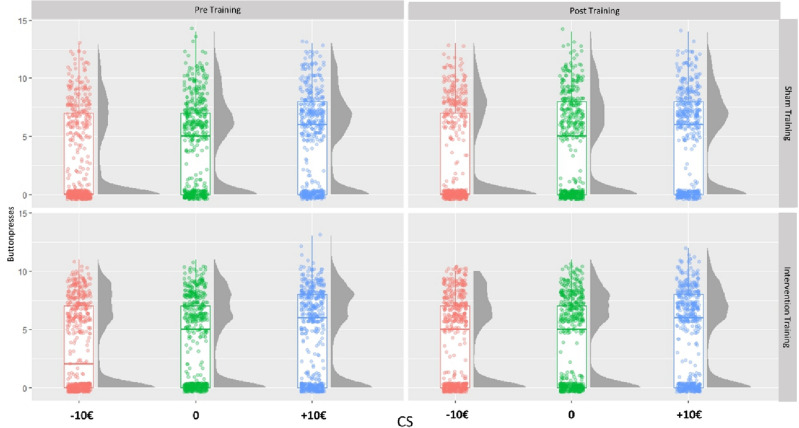
Boxplot/density graph depicting the PIT effect before and after AAT sham/ intervention training. There was a significant interaction effect of CS by time by training, which indicates that the instrumental behavior changed over time due to training. CS = conditioned stimuli.

The GLMM showed significant main effects of CS (*estimate* = 0.530; *SE* = 0.163; *p* = 0.001) and instrumental condition (*estimate* = 1.30; *SE* = 0.055; *p* < 0.001) (*please see *Table 5). The interaction between all fixed effects (CS by training by time by instrumental response) was not significant (*estimate* = 0.330; *SE* = 0.284; *p* = 0.247) (please see supplementary Fig. 1), however the significant interaction between CS by training by time (*estimate* = − 0.459; *SE* = 0.144; *p* = 0.0001) indicates a training-dependent effect of time on the CS-button presses (PIT effect) (*please see *Fig. 4). Simple effects analysis showed a decrease in button presses in negative (*estimate* = − 0.362; *SE* = 0.099; *p* < 0.001) and neutral CS trials (*estimate* = − 0.211; *SE* = 0.091; *p* = 0.021) but not positive CS trials (*estimate* = 0.002; *SE* = 0.089; *p* = 0.985). This points to an increased PIT effect after sham training. The button presses between training conditions also differed after training, here the response rate in negative CS trials was significantly higher after avoidance compared to sham training (*estimate* = 0.6304; *z* = 2.048; *p* = 0.041). In addition, the interaction effect of time by training (*estimate* = 0.448; *SE* = 0.134; *p* < 0.001) as well as time by instrumental condition (*estimate* = 0.280; *SE* = 0.106; *p* = 0.008) was significant. Here, the overall button presses were significantly higher at pre training compared to post training AAT after sham training (*estimate* = 0.448; *z* = 3.360; *p* < 0.001) and button presses in no collect trials were significantly higher before AAT training than after training (*estimate* = 0.280; *z* = 2.641; *p* = 0.008).

**Table 5:** Results of generalized linear mixed model testing the interaction effect of instrumental response, conditioned stimulus, time, and training on button presses in the PIT.

Names	Estimate	SE	95% Exp(B) Confidence Interval			exp(B)	p
			Lower	Upper	z		
(Intercept)	0.866	0.141	1.803	3.135	6.137	2.377	< 0.001
Instrumental condition (collect)	1.301	0.055	3.295	4.094	23.498	3.673	< 0.001
CS	0.530	0.163	1.236	2.337	3.262	1.700	0.001
Time (post training)	− 0.138	0.067	0.764	0.993	− 2.065	0.871	0.039
Training (training condition)	0.406	0.282	0.864	2.609	1.441	1.501	0.150
Instrumental condition (collect) ✲ CS	− 0.129	0.076	0.758	1.020	− 1.701	0.879	0.089
Instrumental condition (collect) ✲ time (post training)	0.280	0.106	1.075	1.628	2.640	1.323	0.008
CS ✲ time (post training)	0.134	0.072	0.993	1.317	1.858	1.143	0.063
Instrumental condition (collect) ✲ training (training condition)	0.016	0.111	0.818	1.261	0.142	1.016	0.887
CS ✲ training (training condition)	− 0.534	0.325	0.310	1.108	− 1.643	0.586	0.100
Time (post training) ✲ training (training condition)	0.448	0.134	1.205	2.034	3.359	1.565	< 0.001
Instrumental condition (collect) ✲ CS ✲ time (post training)	− 0.306	0.142	0.558	0.973	− 2.152	0.737	0.031
Instrumental condition (collect) ✲ CS ✲ training (training condition)	− 0.095	0.151	0.677	1.222	− 0.630	0.909	0.529
Instrumental condition (collect) ✲ time (post training) ✲ training (training condition)	− 0.405	0.211	0.441	1.008	− 1.924	0.667	0.054
CS ✲ time (post training) ✲ training (training condition)	− 0.459	0.144	0.476	0.838	− 3.180	0.632	0.001
Instrumental condition (collect) ✲ CS ✲ time (post training) ✲ training (training condition)	0.329	0.284	0.796	2.424	1.159	1.390	0.247

*CS* conditioned stimulus. *SE* standard error. *exp(B)* odds ratio.

There were no effects of training, time, or an interaction on the proportion of correct trials during the forced choice task (please see supplements, supplementary Table 9 and supplementary Fig. 3).

## Discussion

The goal of the current study was the assessment of an AAT modification training and its impact on the PIT effect. We found a significant PIT effect but there was no impact of Pavlovian CS on approach or avoidance bias operationalized with a joystick AAT. Training to avoid positively valenced CS did not result in a significant change in approach and avoidance propensities. In addition, intervention training did not lead to a decreased magnitude of the PIT effect, but an increased response in negative CS trials. On the other hand, participants showed an increased PIT effect after sham training. To our knowledge, this is the first study that suggests a lack of action bias toward experimentally conditioned cues. On the other side, we also show for the first time that training to approach negatively valenced cues reduced the instrumental behavior towards these cues in the PIT transfer phase.

As expected, we found robust significant PIT effects in accordance with previous reports^15,40,49,50^. Instrumental behavior was enhanced by positive Pavlovian cues, while negative cues constrained instrumental responding. Contrary to our hypothesis, the effect of Pavlovian cue presentation did not translate to approach/avoidance behavior, operationalized by joystick RT in the AAT. We expected the positively valued cues to be approached faster and to be avoided slower and vice versa for negatively valued cues. The lack of significant bias towards the conditioned stimuli might be due to various reasons.

For instance, we are using an irrelevant feature version, i.e., participants respond to a feature related to image orientation (horizontal/vertical) and not to the content of the image per se. Studies have made a strong case for the relevant feature version of the AAT for bias measurement^51,52^.

Although effects seem to be maximized by feature-relevant designs, a design in which subjects are not consciously focusing on the stimuli has been proposed to be a more implicit measure of bias^47,53^. However, as a result, our implicit AAT design might not capture possible bias effects as attention is not drawn to the content of the stimuli. In contrast to the transfer part of the PIT task, the attributes of the stimuli per se might not capture the subjects’ attention in the AAT. In the PIT task, the Pavlovian cues are tiled over the background while subjects are instructed to acknowledge their presence and their associated value but to respond to the instrumental stimuli only. The AAT instructions refer to the orientation of the image only but not to content—in line with previous AAT training studies^54,55^. In addition, there is no reward for behavior in the AAT while participants receive monetary recompense for both the presentation of the Pavlovian cues as well as instrumental responding (although no direct feedback is given after the trials). Another explanation could relate to the operationalization of the response in PIT and AAT i.e., button pressing versus pushing/pulling of a joystick. Conversely, a PIT paradigm that instrumentalized joystick approach/avoidance movements instead of button presses has been established and robust PIT effects have been found^56^. We also found a significant main effect of time that suggests a learning effect reflected by faster RT in the AAT overall.

Furthermore, instructions are an important predictor of approach bias and overall significance was related to individuals awareness of stimuli valences^53^. From an associative stand, AAT bias was attributed to impulsive, automatic processes and training could alter implicit associations towards cues^23^. This view is now challenged by a vast body of research that propose the idea of inferential processes guiding stimulus-action tendencies^57,58^. According to this theory, these tendencies echo learned instrumental significance that translates into goal-driven behavior^57^. In this context, the importance of instructions can be explained by conscious learning of contingencies of the stimuli and alteration of behavior according to task demands. In line with this, hierarchical theories of the impact of cues on behavior propose that expectancy effects play a large role in driving transfer effects^59^. In their study, Hogarth et al.^59^, observed transfer effects to be attenuated by discriminative extinction training (consisting of extinction of instrumental behavior paired with a CS). However, these transfer effects were also abolished by instructing participants that CS were no indicator of the likelihood of outcome. Here it has also been argued that the efficacy of such interventions is based on “instructed extinction” of instrumental probabilities^13^. The intervention training used in this study uses a similar approach, as the CS condition requires the participants to train a new instrumental avoidance behavior. However, factors such as cognitive instructions or operationalization of behavior (button presses versus joystick movements) alter the context in which the Pavlovian CS exerts possible effects on instrumental behavior^60,61^. This context-specificity could explain the lack of effect of Pavlovian conditioning on the initial pre training AAT performance.

After AAT training the approach and avoidance propensities towards the CS changed—overall we saw that negative CS compared to positive CS were avoided slower and approached faster. However, this was surprisingly not driven by training condition and the sham training group showed the same effect. Again, it is not clear if an approach/avoidance bias towards the CS can be established here, the overall increase in RT indicates a strong learning curve of the task demands and in line with the above, subjects might merely focus on image orientation.

When examining the PIT after training, we did, however, find a significant effect. In this case, contrary to our hypothesis, intervention training did not reduce the PIT effect- however, after sham training, the PIT effect was enhanced. The increased PIT slope after sham training did not fall in line with our expectations. As the transfer phase in this paradigm is done in nominal extinction (i.e., without direct rewarding feedback) and the CS presentation during AAT training was not linked to reward, we did not expect the magnitude of the PIT effect to increase in either condition^62,63^. On the other hand, we found button press response towards negative CS to be increased in the group that underwent intervention training compared to sham training. This might indicate a possible training effect in terms of the increased valence of a previously negatively conditioned cue. Since this is not reflected by RT differences between both conditions during post training AAT, we hypothesize that the AAT might not capture the CS effect on instrumental behavior as the PIT paradigm does. Again, the tasks differ in terms of stimulus presentation, instruction, and operationalization of the instrumental action.

As this is a preliminary proof of concept study, the sample size per condition is rather small and we might have not detected possible effects due to low statistical power. Since PIT effects have been proposed to be larger in clinical populations^17,64^, here the effects of training could have a different impact. However, on the other hand, PIT effects have been consistently found in healthy populations^49,50^. As stated above, any possible changes in instrumental behavior might be due to expectancy effects related to instructions or task set-up, however, we have no qualitative or quantitative information from the participants and think that it would be crucial to implement posthoc questionnaires assessing the participants’ subjective judgment on task demands and cognitive strategies. Furthermore, our sample was not balanced in terms of gender distribution, as it contained more women than men. This could potentially affect the generalizability of our results as this imbalance might not accurately reflect prevalence rates in psychiatric conditions, which have been characterized by an altered PIT effect. On the other hand, research has indicated that confounding factors such as gender or age do not influence PIT effects^20^. Our design used monetary reinforcers in instrumental and Pavlovian conditioning, however, different reward types such as e.g., food as a primary reinforcer could elicit stronger or weaker effects on behavior. A study compared the impact of using various reward types and found no difference in PIT effect if their subjective value was matched^65^. In line with this, the monetary rewards might also be valued differently, and future studies should take this into account e.g. by including subjective reward ratings.

In summary, our hypotheses should be investigated in a larger, well-balanced, and clinical sample with i.e., substance abuse or other behavioral patterns marked by conflicts of behavioral goals and values assigned to stimuli.

Taken together we found CS that elicit increased instrumental responding in a PIT task to not affect action bias in an AAT. Sham control training led to a significant increase of the PIT effect. Modification of the PIT effect was seen in that participants that were trained to approach negative CS, showed increased instrumental responses towards those cues in the PIT in contrast to sham training participants. Our findings further contribute to research delineating the underlying mechanism of PIT effects. Some interpretations of our study results refer to the impact of explicit knowledge (i.e., instructions) on how cues energize instrumental behavior. However, further research is necessary to corroborate our findings.

## Supplementary Information

Supplementary Information.

## Data Availability

The datasets generated during and/or analyzed during the current study are available from the corresponding author on reasonable request.
