# Supplementary material for: Modifying Pavlovian-to-instrumental transfer by approach avoidance training in healthy subjects: a proof of concept study
Source: Sci Rep. 2023 Jun 21;13:10074. doi: 10.1038/s41598-023-37083-3 (PMC10284857; doi:10.1038/s41598-023-37083-3)
Supplement: Supplementary file 1 — Supplementary Information. [file 41598_2023_37083_MOESM1_ESM.docx]

**Supplementary Material**

Supplementary table 1. Results of the generalized linear model omnibus (Wald) Chi-Squared testing the pre training AAT.

|  | X² | p |
| --- | --- | --- |
| CS | 3.457 | 0.178 |
| Direction | 2.112 | 0.146 |
| CS ✻ Direction | 0.854 | 0.652 |

Notes. X² = chi-squared; CS = conditioned stimulus

Supplementary table 2. Results of the generalized linear mixed model omnibus (Wald) Chi-Squared testing the effect of response direction, conditioned stimulus, time and training on reaction time in the approach avoidance task (AAT).

|  | X² | p |
| --- | --- | --- |
| Direction | 20.985 | 0.147 |
| CS | 0.5692 | 0.752 |
| Time | 88.714 | 0.003 |
| Training | 0.0489 | 0.825 |
| Direction ✻ CS | 75.775 | 0.023 |
| Direction ✻ Time | 0.0337 | 0.854 |
| CS ✻ Time | 25.172 | 0.284 |
| Direction ✻ Training | 0.4277 | 0.513 |
| CS ✻ Training | 0.4546 | 0.797 |
| Time ✻ Training | 0.0731 | 0.787 |
| Direction ✻ CS ✻ Time | 61.437 | 0.046 |
| Direction ✻ CS ✻ Training | 10.985 | 0.577 |
| Direction ✻ Time ✻ Training | 19.854 | 0.159 |
| CS ✻ Time ✻ Training | 0.6203 | 0.733 |
| Direction ✻ CS ✻ Time ✻ Training | 26.757 | 0.262 |

Notes. X² = chi-squared; CS = conditioned stimulus

Supplementary table 3. Results of the generalized linear model omnibus (Wald) Chi-Squared testing the pre training PIT.

|  | X² | p |
| --- | --- | --- |
| CS | 42.48 | < .001 |
| Instrumental Condition | 637.13 | < .001 |
| CS ✻ Instrumental Condition | 2.81 | 0.245 |

Notes. X² = chi-squared; CS = conditioned stimulus

Supplementary table 4. Results of the generalized linear mixed model omnibus (Wald) Chi-Squared testing the effect of instrumental response, conditioned stimulus, time and training on button presses in the PIT.

|  | X² | p |
| --- | --- | --- |
| Instrumental Condition | 5.521.645 | < .001 |
| CS | 112.678 | 0.004 |
| Time | 42.630 | 0.039 |
| Training | 20.752 | 0.150 |
| Instrumental Condition ✻ CS | 41.247 | 0.127 |
| Instrumental Condition ✻ Time | 69.709 | 0.008 |
| CS ✻ Time | 39.249 | 0.141 |
| Instrumental Condition ✻ Training | 0.0201 | 0.887 |
| CS ✻ Training | 37.549 | 0.153 |
| Time ✻ Training | 112.798 | < .001 |
| Instrumental Condition ✻ CS ✻ Time | 48.496 | 0.088 |
| Instrumental Condition ✻ CS ✻ Training | 0.3971 | 0.820 |
| Instrumental Condition ✻ Time ✻ Training | 37.003 | 0.054 |
| CS ✻ Time ✻ Training | 101.300 | 0.006 |
| Instrumental Condition ✻ CS ✻ Time ✻ Training | 15.237 | 0.467 |

Notes. X² = chi-squared; CS = conditioned stimulus

Supplementary table 5. Results of random effects structure model comparisons with AIC. Anova indicated that the CS+ Direction+ Time model fit best.

| Model | AIC |
| --- | --- |
| intercept | 96271 |
| CS | 96246 |
| Direction | 96158 |
| Time | 95809 |
| CS+ Direction | 96135 |
| CS+Time | 95784 |
| Direction+Time | 95688 |
| CS+Direction+Time | 95665 |

Notes. AIC = Akaike information criterion; CS = conditioned stimulus

Supplementary table 6. Results of random effects structure model comparisons with AIC. Anova indicated that the CS+ Direction+ Time model fit best.

| Model | AIC |
| --- | --- |
| Time | 29400 |
| CS | 29062 |
| CS+Time | 29058 |

Notes. AIC = Akaike information criterion; CS = conditioned stimulus

Supplementary table 7. Parameter estimates of the general linear model testing the interaction effect of response direction and conditioned stimulus on reaction time during the AAT pretest.

|  |  |  | 95% Confidence Interval | |  |  |
| --- | --- | --- | --- | --- | --- | --- |
| Names | Estimate | SE | Lower | Upper | exp(B) | p |
| (Intercept) | 6.54 | 0.01 | 6.5355 | 6.55275 | 695.128 | < .001 |
| Direction (Pull) | -0.013 | 0.01 | -0.0301 | 0.00447 | 0.987 | 0.146 |
| Pavlovian Value  (10€ - 10€) | -0.018 | 0.01 | -0.0387 | 0.00360 | 0.983 | 0.104 |
| Direction (Pull)✻  Pavlovian Value (10€ - 10€) | -0.012 | 0.02 | -0.0544 | 0.03030 | 0.988 | 0.577 |

Notes. SE = standard error; exp (B) = exponential beta/odds ratio

Supplementary table 8. Parameter estimates of the generalized linear model testing the effect of response direction and conditioned stimulus on reaction time during the PIT pretest.

|  |  |  | 95% Exp(B) Confidence Interval | |  |  |
| --- | --- | --- | --- | --- | --- | --- |
| Names | Estimate | SE | Lower | Upper | exp(B) | p |
| (Intercept) | 1.2290 | 0.0234 | 3.265 | 3.58 | 3.418 | < .001 |
| Instrumental Condition (Collect) | 1.1916 | 0.0468 | 3.004 | 3.61 | 3.292 | < .001 |
| CS (10€ - 10€) | 0.3737 | 0.0574 | 1.299 | 1.63 | 1.453 | < .001 |
| Instrumental Condition (Collect)✻ CS (10€ - 10€) | -0.1853 | 0.1147 | 0.663 | 1.04 | 0.831 | 0.106 |

Notes. SE = standard error; exp (B) = exponential beta/odds ratio

*Instrumental condition: Not collecting*


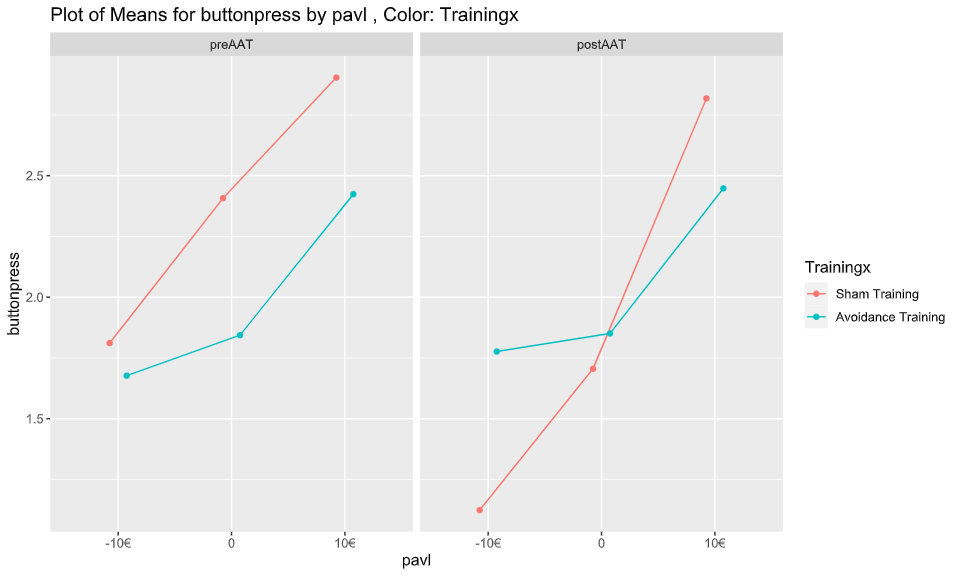


*Instrumental condition: Collecting*


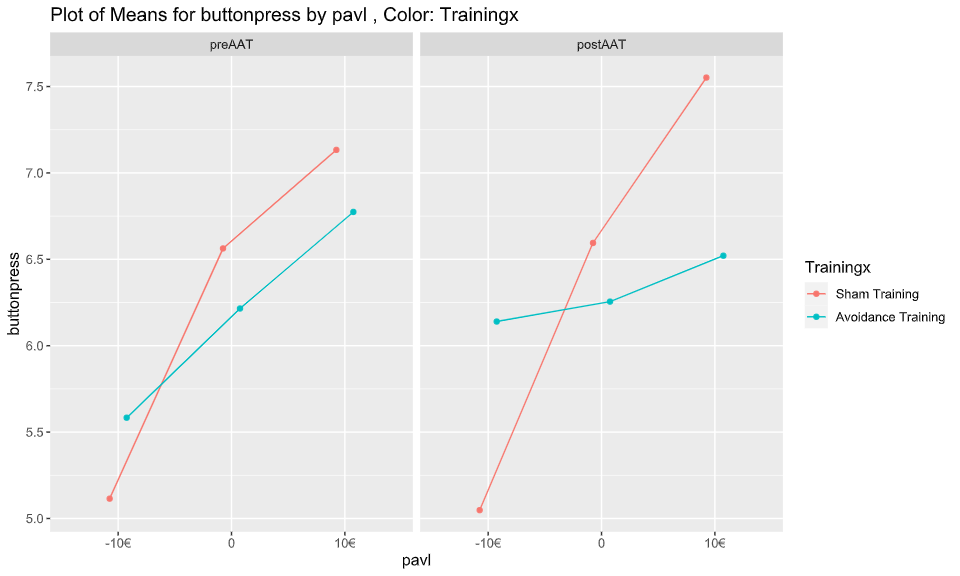


Supplementary figure 1. Effect of sham and avoidance training on PIT button presses divided by instrumental condition (collect/not collect).

Instructions

PIT transfer instructions:

“Keep collecting those ' shells ' that were good and leave the bad ones far away - you'll keep getting 20 cents each for them. If you collect the bad ' shells ', or leave the good ones, you continue to lose 20 cents. In the background you can now see the abstract images. You have learned that each abstract image is assigned to a certain amount of money (win or loss). The monetary value is now randomly credited or subtracted from you half of the time. So, if in the background you can see the abstract image associated with +10 euros, then in half of the cases you can win 10 euros. If in the background you can see the abstract image associated with a loss of 10 euros, then in half of the cases you will lose 10 euros. But this is done automatically by the computer without your intervention. So, focus on winning as much money as possible by collecting shells properly. How much you win or lose in each round, either because of the abstract background image, or because of the shell, is no longer displayed. Your total balance at the end of the experiment will be paid to you. So just keep collecting good shells and keep leaving bad shells. Again, you have 2 seconds to collect a shell or leave it. Press the forward button again to start.”

AAT Instructions:

“Welcome to this joystick task!

We will now show you many individual images on the screen. For each picture you are to move the joystick as fast as possible, so that the picture disappears as quickly as possible. To do this, drag the joystick towards you or push it away from you. Whether you pull the joystick depends on the position of the images:

If the image is higher than wide, pull the joystick towards you. If the image is wider than it is tall, move the joystick away from you.

As soon as you move the joystick completely in the right direction, the picture disappears. Then move the joystick back to the center position and press with the index finger on the trigger of the joystick. The next image is then displayed.

Respond to each image as quickly as possible without making many mistakes. Now let's practice the task with some pictures. Please do not forget: HIGHER THAN WIDE: PULL TOWARDS YOU, WIDER THAN HIGH: PUSH AWAY!”

Query trials

To assess the effect of the training condition over time on the number of correct/incorrect choices during the query trials, we used a logistic GLMM to assess the interaction between time by training. There was no difference between pre and post AAT (*F* = 0.650; *p* = 0.421) as well as nature of training (*F* = 1.209; *p* = 0.280) on forced choice task accuracy. Additionally, the interaction between time and training was insignificant as well (*F* = 1.333; *p* = 0.249), indicating no difference between the conditions over time on forced choice accuracy (*please see supplementary table 2 and supplementary figure 3*).


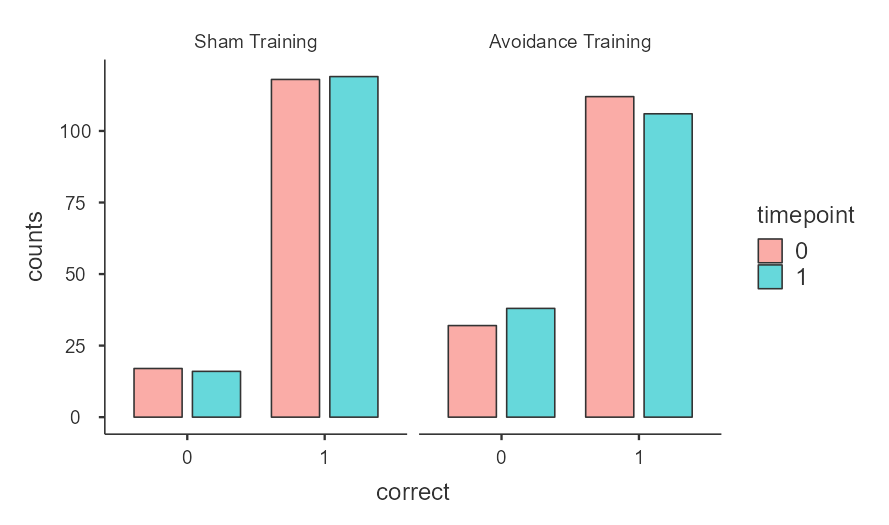


Supplementary Figure 3. Distribution of correct trials pre/post AAT in both sham and avoidance training conditions.

Supplementary Table 9. Results of a logistic GLMM testing the effect of training and time on the proportion of correct choices in the query trials.

|  | F | p |
| --- | --- | --- |
| Timepoint | 0.650 | 0.421 |
| Training | 1.209 | 0.280 |
| Timepoint ✻ Training | 1.333 | 0.249 |
